# Minimally Invasive Management of Adamantinomatous Intraventricular Craniopharyngiomas: A Two-Case Series

**DOI:** 10.7759/cureus.102085

**Published:** 2026-01-22

**Authors:** Fadwa Fliyou, Sidi Mamoun Louraoui, Mounir Rghioui, Faycal Moufid, Abdessamad El Azhari

**Affiliations:** 1 Neurosurgery, Cheikh Khalifa International University Hospital/Mohammed VI University of Sciences and Health, Casablanca, MAR; 2 Neurosurgery, Mohammed VI International University Hospital/Mohammed VI University of Sciences and Health, Casablanca, MAR

**Keywords:** endoscopic resection, intraventricular craniopharyngioma, minimally invasive neurosurgery, stereotactic radiosurgery, third ventricle tumors

## Abstract

Intraventricular craniopharyngiomas (IVCPs) are rare intracranial tumors that pose significant therapeutic challenges due to their deep-seated location, close relationship with critical neurovascular structures, and high risk of treatment-related morbidity. We report two adult cases of adamantinomatous intraventricular craniopharyngiomas, a histological subtype that is uncommon among intraventricular lesions, managed at the Neurosurgery Department of Mohammed VI University Hospital in Casablanca, Morocco. Both patients underwent endoscopic transventricular tumor resection followed by adjuvant Gamma Knife radiosurgery for residual tumor components. Postoperative outcomes were favorable, with improvement of presenting symptoms and preservation of visual, endocrine, and cognitive functions, allowing return to professional activities. After a follow-up period of 24 months in Case 1 and 30 months in Case 2, follow-up imaging demonstrated stable residual lesions without radiological progression. These cases suggest that a minimally invasive strategy combining endoscopic surgery and stereotactic radiosurgery may provide effective tumor control while minimizing neurological and endocrine morbidity in selected patients with intraventricular craniopharyngiomas and are discussed in light of current literature regarding their epidemiology, clinical presentation, radiological features, therapeutic strategies, and prognosis.

## Introduction

Intraventricular craniopharyngiomas (IVCPs) are a rare and anatomically distinct subtype of craniopharyngiomas, defined by their location entirely within the third ventricle [1]. Their deep-seated position and close relationship with the hypothalamus, optic pathways, and surrounding neurovascular structures make surgical management particularly challenging and expose patients to a high risk of postoperative neurological and endocrine morbidity [2,3]. Traditional transcranial or transsphenoidal approaches have long been used to treat craniopharyngiomas; however, in purely intraventricular lesions, these approaches often require extensive manipulation of critical structures and may be associated with significant morbidity, especially when aggressive gross total resection is pursued [3-5]. Recent long-term outcome studies have highlighted an ongoing debate regarding the optimal extent of resection, suggesting that subtotal resection combined with adjuvant radiotherapy may provide comparable tumor control while better preserving neurological and endocrine function [6-8]. In this context, endoscopic transventricular techniques have emerged as minimally invasive alternatives in selected patients, offering improved visualization of the third ventricular cavity, precise tumor debulking, and reduced surgical trauma [1,6,9]. When complete resection is limited by hypothalamic adherence or ventricular floor infiltration, stereotactic radiosurgery represents an effective adjuvant option for controlling residual tumor while minimizing additional surgical risk. Here, we report two adult cases of adamantinomatous intraventricular craniopharyngiomas managed at the Neurosurgery Department of Mohammed VI University Hospital in Casablanca, Morocco, using a strategy combining maximal safe endoscopic transventricular resection and adjuvant Gamma Knife radiosurgery. These cases illustrate a tailored, multimodal approach aimed at achieving durable tumor control while limiting treatment-related morbidity.

## Case presentation

Case 1

**Figure 1:**
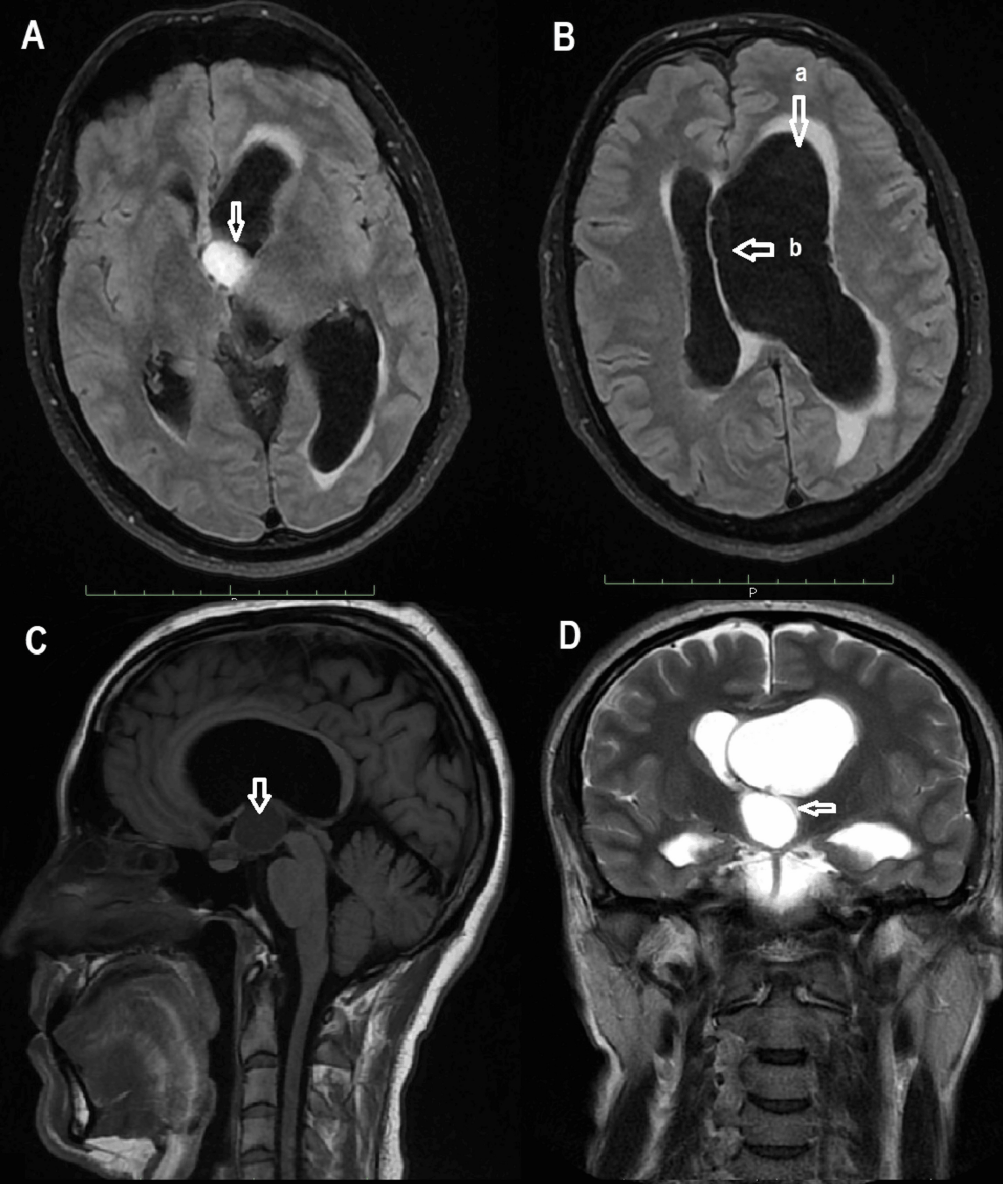
Preoperative brain MRI of case 1 showing a cystic formation with low signal intensity on T1-weighted images (C), high signal intensity on T2-weighted (D) and FLAIR sequences (A), with no contrast enhancement, suggestive of a colloid cyst of the third ventricle, associated with left-predominant hydrocephalus (B) and right subfalcine herniation. A: Preoperative axial FLAIR brain MRI sequence showing a hyperintense cystic lesion within the third ventricle. B: Preoperative axial T2-weighted brain MRI sequence demonstrating left-predominant hydrocephalus. C: Preoperative axial T1-weighted brain MRI sequence showing a hypointense cystic formation without contrast enhancement, suggestive of a colloid cyst. D: Preoperative axial T2-weighted brain MRI sequence illustrating right subfalcine herniation secondary to the mass effect. a: Left-predominant hydrocephalus. b: Right subfalcine herniation.

A 35-year-old male physician, with no significant medical history, presented with expressive aphasia, acute headaches, and an episode of agitation associated with spatiotemporal disorientation. The initial neurological examination was unremarkable except for early-stage bilateral papilledema confirmed by fundoscopic examination. Baseline neuro-ophthalmological assessment showed normal visual acuity and visual fields, and evaluation of the hypothalamic-pituitary axis (including cortisol, thyroid hormones, gonadotropins, and prolactin levels) revealed no abnormalities. Brain MRI demonstrated a well-circumscribed cystic lesion located entirely within the third ventricle, showing low signal intensity on T1-weighted images, high signal intensity on T2-weighted and FLAIR sequences, and absence of enhancement on gadolinium-enhanced T1-weighted imaging, findings that were initially suggestive of a third ventricular colloid cyst. The lesion was associated with predominantly left-sided obstructive hydrocephalus and mild right subfalcine herniation (Figure 1).

**Figure 2:**
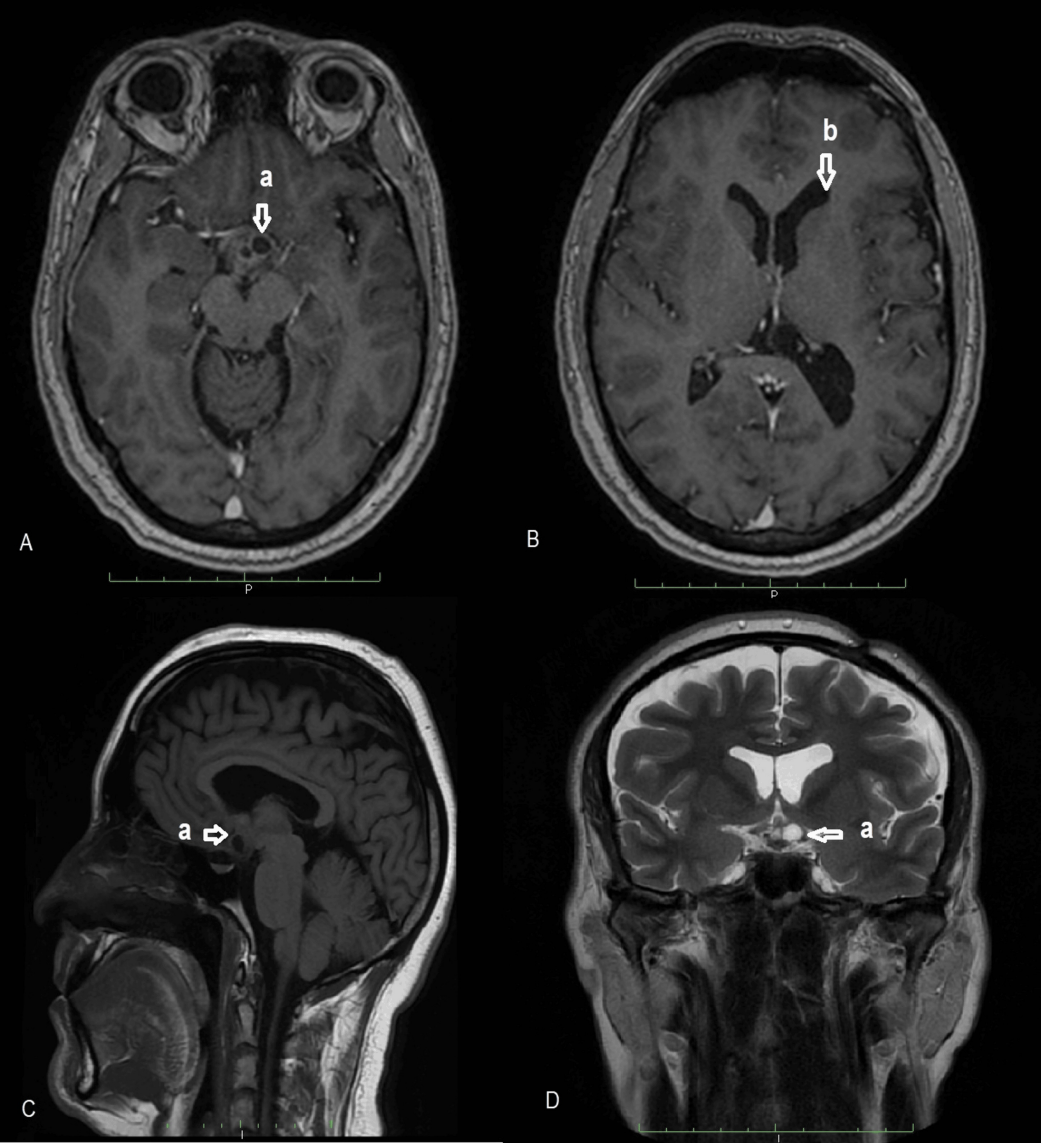
Follow-up brain MRI of case 1 showing residual tumor at the floor of the third ventricle with regression of hydrocephalus. A: Postoperative axial T1-weighted brain MRI sequences showing a residual tumor located at the floor of the third ventricle. B: Postoperative axial T1-weighted brain MRI sequences showing a regression of hydrocephalus. C: Postoperative sagittal T1-weighted magnetic resonance imaging (MRI) sequences reveal a residual tumor along the surgical site. D: Postoperative coronal T2-weighted MRI sequences showing a residual tumor infiltrating the floor of the third ventricle. a: residual tumor. b: regression of hydrocephalus

An endoscopic transfrontal transventricular approach was performed. Intraoperatively, the lesion appeared as a cyst containing yellowish, oily fluid, raising suspicion of an alternative diagnosis. Cyst aspiration and decompression were achieved, followed by partial excision of the cyst wall; complete resection was intentionally avoided due to firm adherence to the ventricular floor. An endoscopic ventriculocisternostomy was performed to restore cerebrospinal fluid circulation. Histopathological examination unexpectedly confirmed an adamantinomatous craniopharyngioma, establishing the final diagnosis. Postoperatively, the patient experienced progressive neurological recovery with resolution of aphasia under medical management. Given the presence of residual tumor and the risk of hypothalamic injury with further resection, adjuvant Gamma Knife stereotactic radiosurgery was performed. A peripheral prescription dose of 11 Gy at the 50% isodose line was delivered, taking into account tumor volume, proximity to critical structures, and histological subtype. At 24 months of follow-up, the patient remained neurologically intact, with preserved visual acuity and visual fields, normal endocrine function, and no cognitive deficits on clinical evaluation, and had resumed full professional activities. Follow-up MRI demonstrated stable residual disease without radiological progression (Figure 2).

Case 2

**Figure 3:**
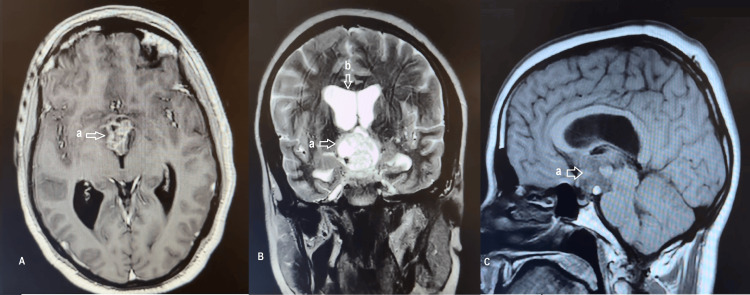
Brain MRI of case 2 showing a suprasellar solid-cystic lesion measuring 32×27×27 mm, causing mild biventricular hydrocephalus and exhibiting hemorrhagic changes. a: suprasellar solid-cystic lesion measuring 32×27×27 mm. b: biventricular hydrocephalus. A: Preoperative axial T1-weighted brain MRI sequences showing a suprasellar lesion. B: Preoperative axial T2-weighted brain MRI sequences showing a suprasellar solid-cystic lesion measuring 32×27×27 mm, causing mild biventricular hydrocephalus and exhibiting hemorrhagic changes. C: Preoperative sagittal T1-weighted brain MRI sequences showing a suprasellar solid-cystic lesion exhibiting hemorrhagic changes.

A 36-year-old male factory worker with a long-standing history of chronic headaches since childhood presented with acute clinical deterioration characterized by sudden worsening of headaches, intractable vomiting, and progressive visual disturbances. Preoperative endocrine evaluation of the hypothalamic-pituitary axis (including morning cortisol, thyroid hormones, and serum electrolytes) showed no evidence of dysfunction. Ophthalmological examination revealed a best-corrected visual acuity of 9/10 in both eyes, with fundoscopic findings of bilateral sectoral papilledema involving the nasal portion of the optic discs. Brain MRI revealed a strictly intraventricular solid-cystic lesion centered within the third ventricle, associated with obstructive hydrocephalus and intralesional hemorrhagic changes. Gadolinium-enhanced T1-weighted sequences demonstrated minimal and heterogeneous enhancement of the solid component, raising suspicion of a craniopharyngioma (Figure 3).

**Figure 4:**
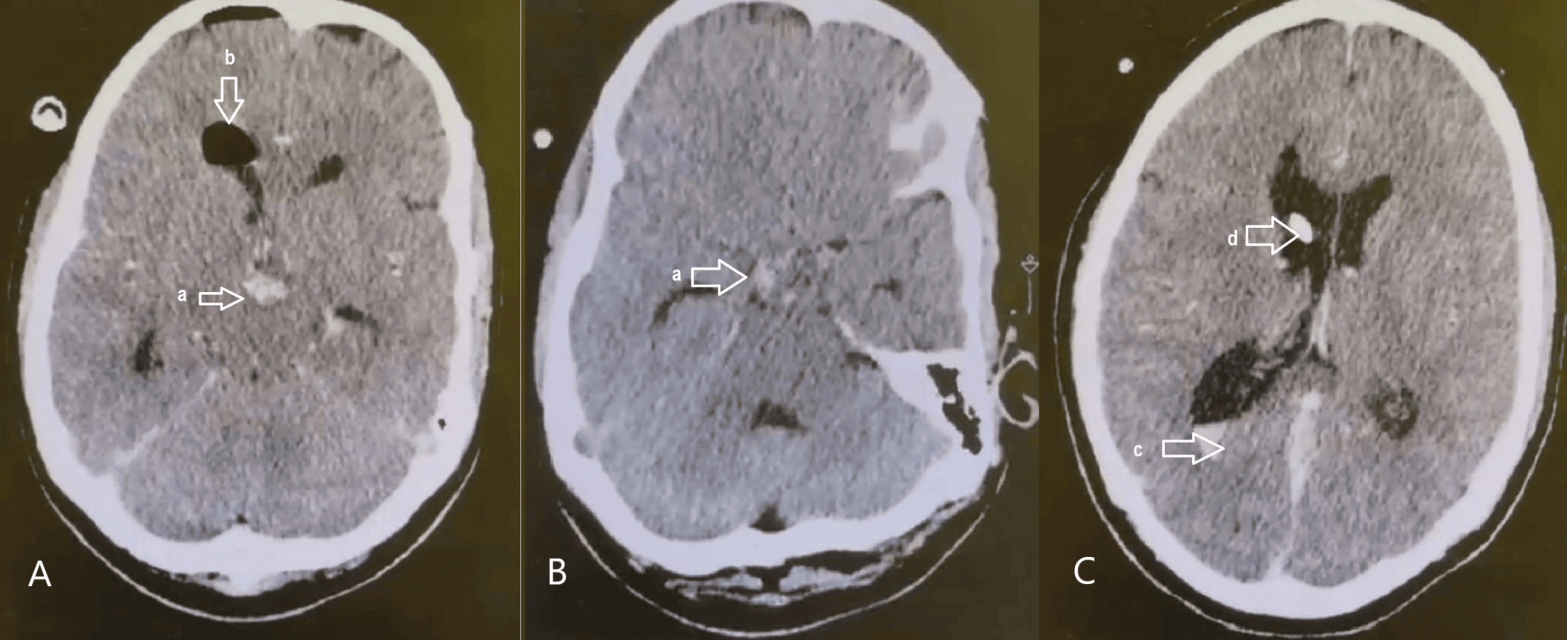
Follow-up brain CT of clinical case 2 scan showing residual tumor with signs of bleeding (a), pneumoventriculism (b), and intraventricular hemorrhage (c). A: Postoperative axial brain CT scan of clinical case 2 showing pneumoventriculism (b) and residual tumor (a) with hemorrhagic changes. B: Postoperative axial brain CT scan of clinical case 2 residual (a) tumor with hemorrhagic changes. C: Postoperative axial brain CT scan of clinical case 2 showing intraventricular hemorrhage (c) with external ventricular drainage catheter (d). a: residual tumor with signs of bleeding. b: pneumoventriculism. c: intraventricular hemorrhage. d: external ventricular drainage catheter.

An endoscopic transfrontal transventricular approach was undertaken. Intraoperatively, the tumor demonstrated dense adherence and focal infiltration of the floor of the third ventricle, with poor visualization of hypothalamic landmarks, rendering safe release of the ventricular floor technically unfeasible and associated with a high risk of hypothalamic injury. Consequently, endoscopic ventriculocisternostomy was not performed. Alternative cerebrospinal fluid diversion strategies, including endoscopic third ventriculostomy via a separate trajectory or lamina terminalis fenestration, were considered but deferred because of distorted ventricular anatomy, active intralesional bleeding, and the elevated risk of hypothalamic damage. The procedure consisted of cyst aspiration and subtotal excision of the cyst wall, followed by placement of a temporary external ventricular drain (EVD), which was maintained for 48 hours to allow controlled cerebrospinal fluid diversion and assessment of hydrocephalus reversibility (Figure 4).

Histopathological examination confirmed an adamantinomatous craniopharyngioma. Postoperatively, the patient developed transient left-sided anisocoria, xerophthalmia, and worsening visual disturbances, followed by temporary diabetes insipidus, all of which were managed conservatively with medical treatment and close monitoring. The EVD was successfully removed after normalization of intracranial pressure, and follow-up imaging demonstrated complete resolution of hydrocephalus without the need for permanent cerebrospinal fluid diversion. Given the presence of residual tumor and the risks associated with further surgical manipulation, adjuvant Gamma Knife stereotactic radiosurgery was performed. A peripheral prescription dose of 11 Gy delivered to the 50% isodose line was selected, taking into account tumor volume, proximity to critical neurovascular structures, and histological characteristics. At 30 months of follow-up, the patient demonstrated objective improvement in visual symptoms, with stabilization of visual acuity and visual fields on clinical assessment, no persistent endocrine deficits, and sustained radiological control of hydrocephalus. The patient resumed professional activities without further neurological complications.

## Discussion

Pathogenesis and epidemiology

Craniopharyngiomas (CPs) are rare central nervous system tumors, with a global incidence estimated between 0.5 and 2 cases per million per year. Intraventricular craniopharyngiomas (IVCPs) represent a small subset, accounting for approximately 4.27% to 8.72% of all CPs [1,2]. IVCPs were first described in 1953 by Dubos et al. and later by Cashion and Young [1,3]. The embryogenesis of intraventricular craniopharyngiomas is thought to involve a developmental anomaly during early brain formation. While adamantinomatous craniopharyngiomas originate from epithelial remnants of Rathke’s pouch and papillary subtypes arise from squamous metaplasia of the pars tuberalis [2], purely intraventricular forms likely result from delayed development of the pial membrane. This delay permits Rathke’s pouch cells to come into direct contact with the neuroectoderm of the developing third ventricular floor, allowing for the possibility of tumor formation entirely within the third ventricle [2,4,5]. IVCPs predominantly affect adults, with a mean age at diagnosis between the third and fifth decades [1,2], consistent with our series (mean age: 35.5 years). Although purely intraventricular lesions have classically been associated with the papillary subtype [6,7], recent studies have demonstrated that the adamantinomatous variant, while uncommon, can also occur in this location [3,7,8], as observed in both of our cases.

Imaging and diagnostic considerations

The diagnosis of IVCPs is frequently delayed due to their slow growth and deep location. MRI remains the imaging modality of choice, providing accurate assessment of tumor composition, ventricular involvement, and hypothalamic displacement [3,7]. Non-enhancing cystic lesions within the third ventricle may initially mimic colloid cysts, particularly in adults, emphasizing the importance of careful interpretation of gadolinium-enhanced sequences and intraoperative findings. Several radiological classifications have been proposed to distinguish strictly intraventricular tumors from pseudo-intraventricular or secondary intraventricular lesions [3,7,9]. These classifications integrate tumor size, shape, consistency, degree of sellar-third ventricle distortion, and hypothalamic involvement and are essential for surgical planning and risk stratification.

Surgical approaches and extent of resection

The management of intraventricular craniopharyngiomas (IVCPs) is particularly challenging because of their exclusive location within the third ventricle and their close anatomical relationship with critical structures, including the hypothalamus, fornix, optic pathways, and surrounding vascular networks [8,9]. In contrast to sellar or suprasellar craniopharyngiomas, intraventricular lesions often lack a distinct and safe dissection plane at the floor of the third ventricle. In both of our cases, intraoperative findings confirmed either firm adherence (Case 1) or direct infiltration (Case 2) of the ventricular floor, making aggressive resection potentially hazardous. Conventional surgical strategies for IVCPs include transventricular approaches via the ventricular roof and lamina terminalis approaches through the floor of the third ventricle. Advances in neuronavigation, endoscopic visualization, and minimally invasive corridors have expanded the surgical armamentarium and improved safety profiles [10]. While transsphenoidal approaches are effective for sellar and suprasellar CPs, they are rarely applicable to purely intraventricular lesions. Endoscopic transventricular techniques have emerged as valuable alternatives, particularly for cystic lesions associated with hydrocephalus, allowing decompression and symptom relief with reduced surgical morbidity [3,11,12]. Traditional surgical approaches for intraventricular CPs may provide wide exposure, but they often require significant brain retraction and manipulation of eloquent neurovascular structures, thereby increasing the risk of postoperative morbidity [13,14]. Given the intraventricular location and ventricular floor involvement observed in our patients, such approaches were considered less favorable. In this context, endoscopic transventricular surgery represented a particularly suitable strategy in both our cases, allowing direct access to the ventricular cavity with enhanced visualization of the tumor-ventricular floor interface while minimizing cortical and subcortical disruption [13-15]. In Case 1, this approach enabled effective cyst decompression and partial resection, with preservation of hypothalamic integrity and favorable neurological recovery. In Case 2, despite successful tumor debulking, the infiltrative nature of the lesion precluded safe fenestration of the third ventricular floor, necessitating temporary cerebrospinal fluid diversion. Historically, attempts at gross total resection (GST) have been associated with high rates of hypothalamic, endocrine, and cognitive morbidity, without demonstrating a consistent improvement in long-term overall or progression-free survival [16-18]. These data support a more conservative surgical philosophy focused on functional preservation rather than radical excision. Comparative analyses suggest that minimally invasive approaches, such as endoscopic transventricular or endonasal strategies, can offer similar tumor control with improved functional outcomes and reduced permanent visual morbidity relative to open surgery [13-15]. However, endoscopic management of intraventricular CPs has inherent limitations. Dense adherence or infiltration of the ventricular floor, as encountered in our second case, may prevent complete resection and expose patients to risks such as hypothalamic injury, cyst recurrence, and postoperative visual or endocrine disturbances [1,12,15]. These limitations underscore the rationale for a multimodal treatment strategy, combining maximally safe endoscopic debulking with adjuvant stereotactic radiosurgery, as applied in both of our patients, in order to achieve durable tumor control while preserving neurological, visual, and endocrine function.

Adjuvant therapies and outcome data

Historically, gross total resection (GTR) was considered the optimal goal; however, growing evidence has questioned the survival benefit of aggressive resection when weighed against the risk of severe and permanent morbidity [19]. Given these limitations, a paradigm shift toward subtotal resection followed by adjuvant radiotherapy has gained acceptance, aiming to balance tumor control with preservation of neurological and endocrine function. Stereotactic radiosurgery (SRS), particularly Gamma Knife radiosurgery, allows precise delivery of high-dose radiation to residual tumor volumes while sparing adjacent critical structures. Available series focusing on intraventricular craniopharyngiomas suggest that combined endoscopic debulking and subtotal resection (STR) provides satisfactory progression-free survival, with lower rates of permanent visual and endocrine morbidity compared with aggressive open resection, although long-term data remain limited. Reported recurrence rates after subtotal resection plus radiotherapy range between 10% and 30%, comparable to those of gross total resection but with improved functional outcomes [12,16,17]. Large retrospective population-based studies support a more conservative surgical philosophy. Schoenfeld et al., in a cohort treated between 1980 and 2009, reported no significant differences in overall survival or progression-free survival between patients undergoing GTR and those treated with subtotal resection (STR) followed by radiotherapy. Importantly, GTR was associated with significantly higher rates of panhypopituitarism and metabolic complications. STR alone, however, was linked to inferior survival outcomes [20]. Similarly, Zacharia et al., analyzing data from the SEER database, demonstrated superior disease control with STR combined with radiotherapy compared with GTR plus radiotherapy in a large patient cohort [21]. Sadashivam et al. further showed comparable long-term visual, endocrine, and hypothalamic outcomes between GTR and STR, with improved tumor control when STR was combined with adjuvant radiation [22]. These findings support the paradigm of maximal safe resection rather than radical excision, particularly for IVCPs with hypothalamic adherence or infiltration. Endoscopic transventricular approaches have gained prominence by offering enhanced visualization, reduced brain retraction, and lower morbidity, especially in cystic lesions associated with hydrocephalus [10,12]. In our cases, the integrity of the third ventricular floor was compromised by tumor adherence or infiltration, precluding safe, complete resection. A strategy of maximal safe endoscopic resection followed by adjuvant Gamma Knife radiosurgery resulted in radiological tumor stability, preserved visual and endocrine function, and favorable clinical outcomes at mid-term follow-up, supporting this multimodal approach in selected IVCPs.

Systemic and targeted therapies

While cytotoxic chemotherapy has not demonstrated efficacy in the management of craniopharyngiomas [12,23-25], recent advances in molecular biology have opened new therapeutic perspectives. Papillary craniopharyngiomas frequently harbor BRAF V600E mutations and have shown meaningful clinical and radiological responses to BRAF and MEK inhibitors, particularly in recurrent or unresectable cases [25,26]. In contrast, adamantinomatous craniopharyngiomas are characterized by activation of the Wnt/β-catenin signaling pathway due to CTNNB1 mutations, and ongoing research is exploring targeted strategies aimed at modulating this pathway [2,27,28]. Although these treatments remain investigational for intraventricular lesions, they represent promising adjuncts in a future multimodal treatment strategy.

Limitations and prognosis

The main limitations of our study include the small sample size and relatively short follow-up duration. Long-term progression-free survival, late endocrine morbidity, and cognitive outcomes require further evaluation through larger, multicenter studies. Nevertheless, our findings support growing evidence that function-preserving strategies combining minimally invasive surgery and radiosurgery may offer optimal outcomes for carefully selected patients with intraventricular craniopharyngiomas.

## Conclusions

The combined use of endoscopic transventricular resection and stereotactic radiosurgery appears to be a safe and effective multimodal treatment strategy for intraventricular craniopharyngiomas. The first case demonstrates that near-total endoscopic debulking followed by adjuvant radiosurgery can achieve satisfactory tumor control with minimal functional morbidity, whereas the second case highlights the technical limitations encountered in the presence of hypothalamic and third ventricular floor infiltration, underscoring the importance of radiosurgery in disease stabilization. This strategy allows maximal safe tumor reduction while minimizing surgical morbidity in anatomically complex locations where radical resection is associated with a high risk of neurological, visual, and endocrine deficits. Nevertheless, the conclusions drawn from this report are limited by the small sample size, relatively short follow-up, and the lack of systematic long-term endocrine, visual, and cognitive evaluations. Future research should prioritize multicenter collaborative studies, long-term outcome analyses, and the integration of emerging molecularly targeted therapies into multidisciplinary treatment algorithms to further optimize patient outcomes.
